# Performance and morphology of several soybean varieties and responses to pests and diseases in South Sulawesi

**DOI:** 10.1016/j.heliyon.2024.e25507

**Published:** 2024-02-02

**Authors:** Abdul Fattah, Idaryani, Herniwati, M. Yasin, Suriani Suriani, Salim, M. Basir Nappu, Sahardi Mulia, Muh Fitrah Irawan Hannan, Heppy Suci Wulanningtyas, Sudjak Saenong, Wanti Dewayani, Suriany, Elisa Winanda, Sri Wahyuni Manwan, Muh Asaad, Warda, Nurjanani, Nurhafsah, Abdul Gaffar, Sunanto, Andi Yulyani Fadwiwati, Maryam Nurdin, Dahya, Andi Ella

**Affiliations:** a Research Center for Food Crops, Research Organization for Agriculture and Food, National Research and Innovation Agency, Jl. Raya Jakarta-Bogor, Km 46, Cibinong, Bogor, West Java, 16911, Indonesia; b Research Center for Horticultural and Estate Crops, Research Organization for Agriculture and Food, National Research and Innovation Agency, Jl. Raya Jakarta-Bogor, Km 46, Cibinong, Bogor, West Java 16911, Indonesia; c Research Center for Agroindustry, Research Organization for Agriculture and Food, National Research and Innovation Agency, Jl. Raya Puspitek, Tangerang Selatan, Banten, Indonesia; d Research Organization for Governance, Economy, and Community Welfare, Jl.Gatot Subroto,No.10. Indonesia; e Research Center for Animal Husbandry, Research Organization for Agriculture and Food, National Research and Innovation Agency, Jl. Raya Jakarta-Bogor, Km 46, Cibinong, Bogor, West Java, 16911, Indonesia

**Keywords:** Soybean, Performance, Characteristics, Population, Level of damage, Pests, Diseases, Seed yield

## Abstract

Soybeans are a commodity that is widely grown by farmers in rainfed rice fields in South Sulawesi. One of the determining factors in increasing soybean productivity in South Sulawesi is the type of variety. The aim of this research was to determine the characteristics, morphology and response to pests and diseases in several soybean varieties planted in rainfed rice fields in South Sulawesi. This research was carried out in Allepolea Village, Maros Regency in 2022 using a Randomized Block Design with 13 treatments and 3 replications. Varieties tested as treatments include: 1) Derap-1, 2) Devon-2, 3) Deja-1, 4) Anjasmoro, 5) Dena-2, 6) Dena-1, 7) Gepak Kuning, 8) Grobogan, 9) Devon-1, 10) Dega-1, 11) Deja-2, 12) Demas-1, and 13) Detap-1. The results showed that of the 13 varieties tested, the highest height was found in Devon-2 (33.67 cm) and Detap-1 (31.67 cm) in the vegetative phase and in the generative phase in Detap-1 (75.53 cm) and Gepak Yellow (74.67 cm). The largest number of branches is in Dena-1 (3.13 branches). The highest nitrogen content was found in Devon-1 (12.64 m2 per g). The largest leaf area was Detap-1 (4.15 cm2) and Gepak Kuning (4.15 cm2). The highest number of stomata was in Dena-1 (42.80 μm) and Deja-1 (44.00 μm). The highest stomata width was found in Gepak Kuning (2.76 μm). The lowest level of leaf damage due to attacks by *Valanga* sp (Acrididae) occurred in Grobogan (6.89 %) and Dega-1 (7.35 %). The lowest level of pod damage due to *Nezara* viridula attack was in Devon-2 (3.56 %) and Dena-2 (3.64 %). The lowest level of leaf damage due to *Phaedonia inclusa* attack occurred in Dega-1 (4.37 %), Dena-2 (4, 12 %), and Grobogan (4.69 %). Seed damage due to *Cercospora* sp attack was lowest on Dena-2 (0.81 %). The highest seed yield was in Dena-2 (3.78 t ha-1) and the lowest in Anjasmoro (1.93 t ha-1) and Deja-2 (2.02 t ha^−1^).

## 1 Introduction

Soybeans are a source of vegetable protein that is popular with Indonesian people. The annual need for soybeans in Indonesia is around 2,993,104 tons per year. Meanwhile, soybean production only reaches an average of 337,985 t per year, so soybeans have to be imported around 2,595,199 t per year [1]. Soybean productivity in Indonesia is only around 1.60–1.85 t ha-1 (Ministry of Trade, 2022). One of the reasons for the low productivity of soybeans is that the variety used by farmers (Anjasmoro) only has a productivity of around 1.65 t ha-1 [2]. Furthermore [3], stated that the seed yield achieved by Anjasmoro was only 1.52 ha-1 and lower than Grobogan (1.78 ha-1) [4]. the seed yield per plant obtained from the Anjasmoro variety was lower (13.24 g per plant) than Argomulyo (14.50 g per plant). The Anjasmoro variety, apart from having low productivity, is also susceptible to attacks by the armyworm *Spodoptera litura*. According to Ref. [5], in the vegetative phase of soybeans, the level of leaf damage due to *S.litura* pest attacks was highest in the Anjasmoro variety (30.67 %) and lowest in the Grobogan variety 23.95 %, likewise in the generative vase, the highest level of soybean leaf damage in Anjasmoro (28.90 %) and the lowest in Grobogan 22.41 %.

To overcome this problem, one way is to introduce superior soybean varieties which have a high seed yield potential of above 2.0 t ha-1. The research results show that there are several superior soybean varieties that have been produced by Indonesian researchers which can achieve a seed yield potential of between 2.0 and 3.0 t ha-1 include Grobogan (2.77 ha-1), Detap-1 (2.70 t ha-1), Derap-1 (2.82 ha-1), Gepak Kuning (2.86 ha-1, Devon-1 (Dega −1 (3.82 t ha-1) [5]. Apart from having high productivity compared to Anjasmoro, this variety is also more resistant to pests. According to *(*[6], the highest population level of the *Nezara viridula* pest was found in the variety Deja-1 (1.42 individuals per plant-1) and Dena-1 (1.17 individuals per plant-1) while the lowest was in the Detap-1 variety (0.83 %). According to *(5)*, the Dega-1 variety is resistant to pod borer pests while the Argomulyo variety only had a pod damage rate of only around 13.11 %. [7], the Derap-1 and Detap-1 varieties are resistant to soybean pod sucker (*Riptortus linearis*) and soybean pod borer (*Etiella zinckenella*)

Morphological characteristics and form are factors that influence the level of productivity and the level of damage caused by pests and diseases. According to Ref. [8], the types of pests that cause a lot of damage to soybeans include the armyworm *S. litura*, the beetle beetle *R. linearis*, the green ladybug *N. viridula*, the pod borer *E. zinckenella*, the whitefly *Bemisia tabaci*, the soybean beetle *Phaedoniainclusa*. Each variety has a different response to pest attacks and plant diseases.

Therefore, to know the characteristics and morphology of each variety grown in an area, it is necessary to carry out further research on several soybean varieties. The same thing was stated by Ref. [9], to determine the superiority of a variety, efforts need to be made to identify and analyze its superiority for commercial cultivation. Furthermore [10], to determine the adaptability of a soybean variety to climate stress and response to pests and diseases, site-specific characteristic and morphological adaptation tests are needed. Meanwhile, according to Ref. [3], to determine the characteristics and adaptability of soybeans to a specific area in a certain period, an adaptation test is needed.

This research aims to determine the characteristics, morphology and response to main pests and diseases as well as the highest seed yield of 13 superior soybean varieties tested in rainfed rice fields. The expected results are to provide information about the characteristics and morphology as well as seed yield and the level of damage due to attacks by the main pests of each soybean variety studied. Information data from the results of this research will be used as recommendation material for the use of soybean varieties that are suitable for rainfed rice fields in South Sulawesi.

## 2 Materials and methods

Morphological performance research on several superior soybean varieties was carried out in Allepolea Village, Lau District, Maros Regency from April to December 2022. The results of soil analysis showed that the soil characteristics at the research location included a dusty clay loam texture, high Cation Exchange Value (CEC), and medium C/N value (Soil Laboratory, Ministry of Agriculture). Meanwhile, the temperature and humidity during the research period ranged from 26.4 to 27.9 and humidity ranged from 76 to 90 %. This research used a Randomized Block Design with 13 treatments and 3 replications. Varieties as treatments tested include: 1. Derap-1, 2. Devon-2, 3. Deja- 4. Anjasmoro, 5. Dena-2, 6. Dena-1, 7. Gepak Kuning, 8. Grobogan, 9 Devon-1, 10. Dega-1, 11. Deja-2, 12. Demas-1, and 13. Detap-1. This soybean variety was chosen because it is a new superior soybean variety in Indonesia and has specific advantages that vary according to farmers' needs. The thirteen soybean varieties are soybeans with high potential and average yields and are resistant to pests and disease. Each has its advantages with the following explanation: Grobogan has a short harvest period, namely around 76–85 days and also has a high protein content. Yellow Gepak is suitable as a raw material for tofu because it has a high yield of tofu. This soybean variety is adaptive to various land agroecosystems. Dena 1 and Dena 2 are shade-tolerant soybeans which are suitable for planting in young stands of plantations and industrial forests (<4 years) as well as intercropping with corn or cassava plants. This soybean is early maturing with a harvest age of 78 days. Deja 1 and Deja 2 are soybeans that are very tolerant to water saturation stress from the age of 14 days until maturity. Harvest age 79 days. Devon 1 and Devon 2 are soybeans with high isoflavone levels (1097–2200 μg per g) compared to other soybean varieties. Derap 1 is black soybeans as raw material for soy sauce. Dega 1 a early maturing soybean that can be harvested 70–73 days after planting, and is resistant to lodging. Demas 1 is an adaptive soybean for dry, acidic land, best planted at an altitude of 600 m above sea level. Detap 1 is a soybean that is resistant to pod splitting and has a maturity of 78 days [11].

This variety was planted in plots measuring 3 m × 5 m with a spacing of 15 cm × 50 cm. Planting 2 soybean seeds per planting hole was done with a hammer. NPK fertilizer application at a dose of 250 kg per ha was carried out at the age of 21 days after planting. Fertilization was only done once by digging into the soil.

### 2.1 Measurement of Plant chlorophyll value

Measuring leaf chlorophyll in the field uses a SPAD chlorophyll meter. Each leaf sample whose chlorophyll content is measured is clipped to the sensor part of the tool. SPAD sensors are placed at the base of the leaf, middle and tip of the leaf [12] The parameters measured are: leaf chlorophyll content, leaf nitrogen content, temperature and humidity.

### 2.2 Soybean leaf surface area

Leaf area measurement uses Leaf Area Meter (LAM). One plant was taken from each variety, then taken to the laboratory for leaf area measurements. The leaf to be measured is inserted into the LAM, then the leaf is pulled slowly. After the leaf comes out of the LAM tool, the leaf area value automatically appears on the tool display. To save the data, press the save button.

Soybean leaf area was measured using a Leaf Area Meter (LAM) with Specifications Model LAM-A Unit Millimeter, Square Centimeter Precision ± 2 %, Resolution 0.01 cm Measuring Length≤1000 mm, Measuring Width ≤160 mm, Thickness ≤8 mm Data Capacity ≥1000 Groups, Packing Size(W*D*H) 390*230*220 mm Gross.

### 2.3 Parameters observed in this study

Parameters observed were: plant height, number of branches, leaf shape, leaf area, humidity and temperature, chlorophyll and nitrogen content in leaves, the level of humidity and temperature on the leaves, number of stomata, a width of stomata, length of stomata, and shape of stomata on soybean leaves, pod length, number of seeds per pod, number of pods, number of filled pods and empty pods per plant, the color of the pods, number of pods per node, the number of pest populations and the level of damage to soybean leaves and pods, seed hilum color, production of soybean stover, aspects of processing soybeans into products, aspect of market opportunities, the weight of 100 seeds, and seed yield.

The rate of damage to the leaves is calculated based on formula 1 [13]:

(1) $$I=\frac{\sum_{i=0}^{z}\left(n1\;xv1\right)}{ZxN}x\;100\%$$

I: intensity of damage

n: the number of leaves with a $v_{i}$ scale

N: number of leaves observed.

Z: the higher $v_{i}$.

Scale value, $v_{i}$:

0: no damage on leaves.

1: leaf damage >0 %–20 %

3: leaf damage >20 %–40 %

5: leaf damage >40 %–60 %

7: leaf damage >60 %–80 %

9: leaf damage >80 %–100 %

### 2.4 Statistical analysis

Data were analyzed by analysis of variance (ANOVA) using IPM SPSS Statistic 24. The comparison of mean components of growth, yield, and leaf damage intensity, and other parameters were made using the Duncan test at a 5 % probability level.

## 3 Results and discussion

### 3.1 Plant height and number of branches per plant

Plant height and number of branches are one of the factors that influence the high and low seed yields. According to Ref. [14], seed weight is positively correlated with the number of plant branches. In Table 1, it can be seen that all the varieties tested had differences in plant height both at 30 days after planting (vegetative phase) and at 60 days after planting (generative phase). However, of all the varieties tested, the ones that gave the best growth with the highest plant height were Devon-2 and Detap-1 in the vegetative phase. Meanwhile, in the generative phase, the best growth was found in the varieties Detap-1, Gepak Kuning, Devon-2, Deja-1, Dena-1, and Devon-1. This is in accordance with [15], that the plant height achieved by the Detap-1 variety at the age of 30 days after planting was around 30.43 cm. Likewise [16], stated that the growth of the Gepak Kuning variety was lower with only a plant height of 63.83 cm lower than Dena-1 (79.81 cm).

**Table 1:** Plant height and number of branches at the age of 30 and 65 days after planting (DAP).

Type of variety	Plant height (cm)		Number of Branches	
	30 DAP	65 DAP	30 DAP	65 DAP
Derap-1	25.06BCE	46.00a	2.93 ab	3.67abc
Devon-2	33.67f	70.73cdef	2.47 ab	3.47abc
Deja-1	28.40cde	73.07 def	1.67 ab	3.13 ab
Anjasmoro	29.57de	73.47de	1.93 ab	3.13 ab
Dena-2	20.80a	49.33a	1.73 ab	3.47abc
Dena-1	26.67bcd	70.60cdef	3.13b	3.80BCE
GepakKuning	26.60bcd	74.67f	2.27 ab	4.00c
Grobongan	29.93de	60.67BCE	2.47 ab	3.00a
Devon-1	28.13bcd	66.33cdef	1.87 ab	3.20 ab
Dega-1	27.93bcd	54.60 ab	2.60 ab	3.33 abc
Deja-2	24.80b	62.80bcd	1.87 ab	3.40 abc
Demas-1	25.00BCE	63.67bcde	2.00 ab	3.53abc
Detap-1	31.67ef	75.53f	1.60a	3.27abc

*The numbers in the same column followed by same letter are not significantly different according to Duncan's test at the 0.05 level.

Of all the varieties tested, only 2 varieties had significant differences in the number of branches achieved per plant, the others were almost the same at the age of the plants 30 days after planting. The same thing happened at 60 days after planting, of all the varieties tested, only 2 varieties were significantly different, the others were almost the same. Number of branches per plant, in the vegetative phase 30 days after planting, it turns out that only the Dena-1 variety gives the highest number of branches per plant (3.13 branches) while the others only have 1–2.93 branches. However, after the plant is 65 days after planting, the plant has entered the generative phase. The number of branches per plant for all varieties has generally reached 3.0–3.80 branches per plant. This is in accordance with [11], the number of branches per plant achieved by all Dena-2 varieties, Dena-1, Devon-1, Derap-1, Derap-1, Detap-1, Deja-2, and Deja-1 give 3–4 branches per plant.

### 3.2 Leaf shape, leaf area, humidity, temperature, chlorophyll, and nitrogen content in leaves

#### 3.2.1 Leaf shape

**Fig. 1:**
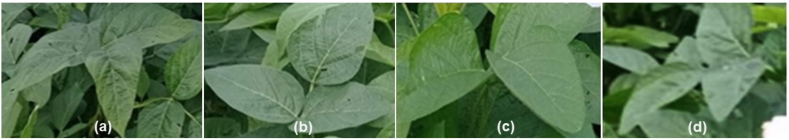
Leaf shape of several soybean varieties in South Sulawesi: (a) Pointed leaf (Grobogan); (b) Tapered oval (Devon-2); (c) Rounded ovals (Devon-1), and (d) Triangular leaf (Dena-2).

There are 4 shapes of soybean leaves in the 13 test varieties, namely pointed leaf, tapered oval, rounded oval, and triangular leaf shape. Soybean varieties that have tapered oval leaves include Devon 2 and Grobogan Kuning. Meanwhile, the pointed leaf shape is found in Grobogan, the rounded oval leaf shape in Devon-1, and the triangular leaf shape in Dena-2 (Fig.1). This is in accordance with [17], the Demas-1 variety has oval leaves, the Anjasmoro variety has oval leaves, while the Grobogan variety has sharp leaves. Soybean varieties that have oval leaves usually have a higher level of pest attacks than those with pointed leaves. Varieties with pointed leaves generally have lower levels of attack by leaf-eating pests than varieties with oval leaves. According to Ref. [18], the Grobogan variety with sharp leaves had a lower level of leaf damage caused by S. litura (23.69 %).) compared to the Anjasmoro variety which has oval leaves (32.68 %). One of the reasons is that soybean leaves which are pointed generally allow more light to enter or penetrate the ground so the temperature is slightly higher and the humidity is slightly lower than oval soybean leaves. This is in accordance with *(*19*)*, high temperatures causing low humidity will trigger the development of pests. Meanwhile, the disadvantage is that the oval shape is usually more preferred by insect pests for laying eggs compared to the pointed shape [11].

#### 3.2.2 Leaf area, leaf greenness, and leaf nitrogen content

**Fig. 2:**
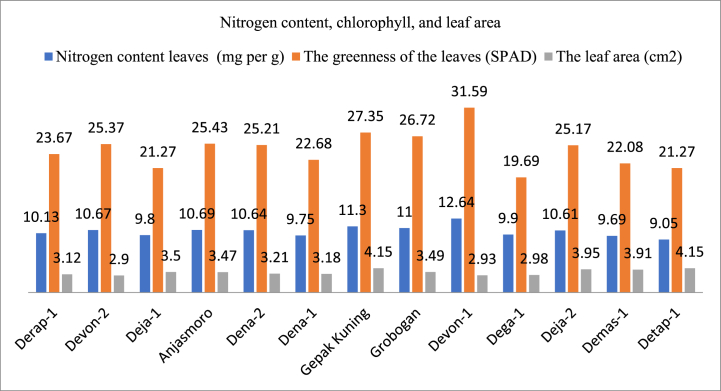
Nitrogen content and level of greenness in leaves and leaf area of several soybean varieties in South Sulawesi.

One of the important plant characteristics is the leaves, regardless of the size, shape, and position of the leaves of Fig. 2 shows that there is a linear relationship between nitrogen content and leaf greenness, especially at nitrogen levels above 10 mg per g. The higher the level of greenness, the higher the nitrogen content found in Devon-1, Gepak Kuning, Grobogan, Anjasmoro, Devon-2, Dena-2, Deja-2 and Derap-1. In the figure it can be seen that the highest leaf area was obtained in the varieties Gepak Kuning and Detap-1 are 4.15 cm2, while the lowest leaf area is obtained from the varieties Devon-1 (2.90 cm, Devon-2 (2.93 cm), and Dega-1 (2.98 cm). One of the important plant characteristics is the leaves. Whatever the size, shape and position of the plant's leaves. Plants obtain carbon through photosynthetic organs, namely leaves [19]. Leaf area is directly related to plant metabolism, especially photosynthesis and respiration [20]. Leaves with a larger size have a higher chance of capture light. Light intensity has a significant effect on the photosynthesis rate of plants. Higher light intensity generally results in a higher photosynthesis rate [21].

Varietal differences had no significant effect on leaf area. Different soybean varieties are sensitive to changes in the environmental conditions in which they grow [22]. [23] stated that the increase in leaf area was caused by plants trying to adapt to avoid lack of light in shady environments. Leaf area is influenced by genotype and environment, where leaf area supports plant growth, biomass production, and vigor because photosynthesis produces assimilation for plant life needs [24]. Plants use assimilate for cell division and enlargement so that their leaves expand. The increase in leaf area is influenced by the lateral meristem which is located at the edge of the widened young leaves [25]. Plants that have the widest leaf area at the beginning of their growth will grow and develop faster because they have higher photosynthesis. The Gepak Kuning and Detap-1 varieties produce leaves with a wider total leaf area (Fig. 2) but produce lower seeds than the Grobogan, Dena-2 and Devon-2 cultivars (Table S2). Leaf area and leaf shape are related to morphological traits that influence soybean yield. Genetically, soybean plants with broad leaves tend to produce fewer seeds, while soybean plants with narrow, pointed leaves tend to produce more seeds [26].

The level of greenness of leaves is an indicator to determine the chlorophyll content in leaves. Chlorophyll is the most important component of chloroplasts for photosynthesis and also has a positive relationship with the rate of photosynthesis [27]. According to Ref. [28], the correlation coefficient between the optimal spectral index and the chlorophyll content of soybeans is above 0.5, indicating a positive correlation between chlorophyll content and photosynthesis.Nitrogen (N) is very important in the vegetative phase and is the main nutrient most needed for plant growth and development. Plants that grow with sufficient N levels become greener. In addition, N is an essential nutrient and its availability is highly correlated with plant growth, yield, and response [29].

Varietal differences do not have a significant effect on the level of leaf greenness and nitrogen content of soybean plants. The highest level of leaf greenness and nitrogen content were obtained in the Devon-1 variety, namely 31.59 and 12.64 respectively. This shows that the Devon-1 variety has a better level of leaf greenness and nitrogen content than other varieties. The level of greenness of the leaves is positively correlated with the chlorophyll content contained in the leaves. The chlorophyll concentration in several varieties can vary and this is influenced by several factors such as genes, water and plant age. Chlorophyll concentration will generally increase in the early growth phase or vegetative phase and will decrease in the aging phase [30]. From the data on leaf greenness and nitrogen content, it can be seen that there is a positive correlation, where the higher the level of greenness, the higher the N value. According to Ref. [31], leaf nitrogen content is one of the factors that can influence leaf chlorophyll content. Leaf chlorophyll content provides useful information regarding the photosynthetic capacity of leaves as determined by the maximum carboxylation level [32]. Nitrogen content in leaves is closely related to chlorophyll formation [33], explaining that nitrogen availability is an important factor in controlling the synthesis of photosynthetic pigments in plant leaves, and there is an important direct relationship between nitrogen supply and chlorophyll concentration in plants. Nitrogen deficiency can slow down chlorophyll formation, reduce photosynthesis rates and disrupt plant metabolic activities [34]. In line with the opinion of [35] which stated that the nitrogen content in leaves is very important structurally and as a metabolic component, they found that the proportion of leaf N allocated to cell walls increased with increasing leaf dry mass per unit area.

The negative impact of the level of green leaves and high nitrogen content will affect the level of damage to plants including leaves due to pests and diseases. Pest insects generally prefer to lay their eggs and feed on green plants that contain high nitrogen. According to Ref. [36], cotton that has green flower petals is preferred by cotton beetles to lay their eggs and breed there.

#### 3.2.3 The level of humidity and temperature of the leaves

**Fig. 3:**
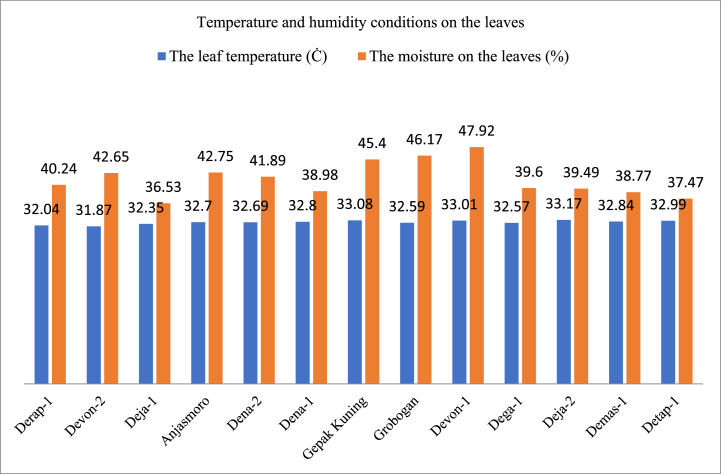
Temperature and humidity conditions on the leaves of several soybean varieties in South Sulawesi.

Fig. 3 and Table 2 show that the data varies and high leaf humidity is not always accompanied by large stomata width. The Devon 1, Grobogan, and Anjasmoro varieties have higher leaf humidity than other varieties, but their stomata width is smaller than other varieties with lower leaf humidity. This is the plant's attempt to conserve water in its leaves. The Demas 1 variety has low air humidity but the width of the stomata is not too small. The Gepak Kuning, Devon 2 and Dena 2 varieties showed higher leaf humidity than the other varieties, as well as larger stomata width. Likewise, the Detap 1 and Deja 2 varieties showed the same value. The Dena 1 and Detap 1 varieties have relatively lower leaf humidity compared to other varieties and have relatively lower stomata width and efforts to increase water efficiency in the leaves. For leaf temperature parameters, in general almost all varieties that have higher leaf temperatures have smaller stomata width, and vice versa, only the Gepak Kuning and Deja 1 varieties show different patterns. Stomatal conductance is strongly influenced by leaf humidity, where under water stress conditions, humidity plays a role in regulating plant physiological responses, especially the level of stomatal opening in leaves [37].Studies report that artificial humidity through watering around the leaves triggers the opening of the stomata which allows plants to more efficiently take up nanoparticles in the form of fertilizers or pesticides so that farmers do not overuse them and pollute the environment [38]. Humidity affects photosynthesis and plant survival under conditions of heat and drought stress [39]. Plant stress due to heat conditions causes chloroplast damage and inactivates rubisco activase (RCA), reduces the important role of chloroplasts, and affects photosynthetic efficiency [40]. Photosynthesis, transpiration rate, and stomatal conductance depend on leaf temperature and can reduce the observed plant photosynthesis rate by 21 % and 63 % and the transpiration rate will increase when leaf temperature increases above 25 °C [41].

**Table 2:** Number of stomata, a width of stomata, and length of stomata on leaves.

Type of variety	Stomata on Soybean Leaves		
	Number of Stomata	Width of stomata (μm)	Length of stomata (μm)
Devon 2	35.87abc	2.69b	4.20a
Dena 2	40.33BCE	2.48 ab	4.12a
Anjasmoro	32.07a	2.32 ab	4.16a
GepakKuning	34.40 ab	2.	4.20a
Detap 1	37.53abc	2.15a	4.21a
Devon 1	39.87abc	2.32 ab	4.11a
Dega 1	40.60BCE	2.48 ab	4.29a
Demas 1	34.33 ab	2.39 ab	4.23a
Deja 1	44.00c	2.32 ab	4.09a
Grobogan	39.00 abc	2.08a	4.25a
Dena 1	42.80c	2.27 ab	4.16a
Deja 2	39.67abc	2.39 ab	3.96a
Derap 1	37.47abc	2.45 ab	4.36a

*The numbers in the same column followed by same letter are not significantly different according to Duncan's test at the 0.05 level.

### 3.3 Number of stomata, a width of stomata, length of stomata, and shape of stomata on soybean leaves

#### 3.3.1 Number of stomata, a width of stomata, length of stomata on soybean leaves

In Tables 2 and it can be seen that the results of observing the number of stomata, stomata width and leaf stomata length varied between the varieties observed. The observation results showed that the number of stomata was highest in the Deja 1, Dena-1, Dena-2, and Dega-1 varieties. The number or density of stomata is influenced by various factors including the photosynthetic capacity of soybean plants [42]. Another thing that influences the density and size of stomata is plant physiological activities such as photosynthesis and transpiration as well as influencing plant metabolism, nutrient absorption and accumulation of organic matter [43,44]. In contrast to observations of leaf stomata width, the Gepak Kuning variety has the highest stomata width which is significantly different from other varieties. The differences in stomata density and size that are influenced by each variety are greatly influenced by various factors such as relative humidity, temperature, carbon dioxide concentration in the atmosphere, light intensity, and plant hormones [45]. The hormone auxin is a plant hormone that regulates stomata [46,47]. Light plays an important role in stomata development. Plants grown in low light conditions had significantly reduced numbers of stomata compared to plants grown in sufficient light conditions. Increasing light intensity can increase the density and size of stomata [48]. The density and size of plant stomata decreases as the concentration of carbon released in the atmosphere increases [49,50].

### 3.4 Pod length, number of seeds per pod, number of pods, number of filled pods, and empty pods per plant

Pod length, number of seeds per pod, number of filled pods, and number of empty pods are factors that influence soybean seed yield. According [14], the number of filled pods and the number of empty pods is positively correlated with seed yield. In Table 3 it can be seen that there are differences in the characteristics of the pods of the varieties tested. In the table, Dega-1 has the highest pod length (5.03 cm) but this has not been compared with other observed characteristics such as the number of seeds per pod, pods per plant, number of fruiting pods per plant, and the lowest pod length in Gepak Kuning (3.11 cm). The number of seeds for each variety is almost the same, around 2–3 seeds per pod. The highest average number of seeds was found in the Dena-2 variety, namely 2.77 seeds per pod and the lowest average was found in the Dega-1 (2.20 seeds per pod) and Gepak Kuning (2.22 seeds per pod) varieties. The highest number of pods was produced by the Demas-1 variety (108.33 pods per plant) with the highest average number of filled pods and the variety with the lowest number of pods was produced by the Grobogan variety (34.53 pods per plant). Soybean production is greatly influenced by the number of pods produced. The variety with the highest number of fruiting pods was produced by the Demas-1 variety (104.87 pods per plant) and the lowest number of fruiting pods was produced by the Grobogan variety (33.47 pods per plant). Pod length is a genetic variability that can be measured and is a consideration in soybean breeding. The morphological characteristics that are formed are the interaction of plants with the environment, water availability, and plant nutrients available optimally during the photosynthesis process. Pod length is positively correlated with pod blast susceptibility [14]; Sclerenchyma along the length of the pod as well as elongated exocarp cells are important factors in the emergence of legume pods [51]. The low amount of photosynthate received by plant organs causes the weight of photosynthate storage organs, especially production organs such as seeds, to be low. A high photosynthesis rate indicates that there is a sufficient supply of nutrients to support the growth of plant seeds [32].

**Table 3:** Pod length, number of seeds per pod, number of filled pods and empty pods per plant.

Type of Variety	Pod length (cm)	Number of seeds per pod (seed)	Number of pods per plant	Number of filled pods per plant	Number of empty pods per plant
Derap-1	4.30bcd	2.48abcd	52.87 ab	50.80 ab	2.07abcd
Devon-2	4.35cde	2.51abcd	64.93 ab	63.33 ab	1.60 ab
Deja-1	4.29bcd	2.69cde	58.80 ab	55.00 ab	3.80de
Anjasmoro	4.34bcde	2.46abcd	71.27abc	68.60abc	2.67abcd
Dena-2	4.57 def	2.77de	80.40BCE	76.13BCE	3.67cde
Dena-1	4.77 def	2.58bcde	73.73 abc	70.07 abc	4.27e
GepakKuning	3.11a	2.22 ab	71.53abc	68.20 abc	3.33bcde
Grobongan	4.85ef	2.37 abc	34.53a	33.47a	1.07a
Devon-1	4.25bcd	2.51abcd	69.13 abc	66.60abc	2.53abcd
Dega-1	5.03f	2.20a	41.27 ab	39.67 ab	1.60 ab
Deja-2	3.91BCE	2.36 abc	71.27abc	68.67abc	2.33abcd
Demas-1	3.82b	2.45abcd	108.33c	104.87c	3.47bcde
Detap-1	4.90f	2.45abcd	48.67 ab	46.87 ab	1.80 abc

*The numbers in the same column followed by same letter are not significantly different according to Duncan's test at the 0.05 level 3.5.

### 3.5 The color of the pods

**Fig. 4:**
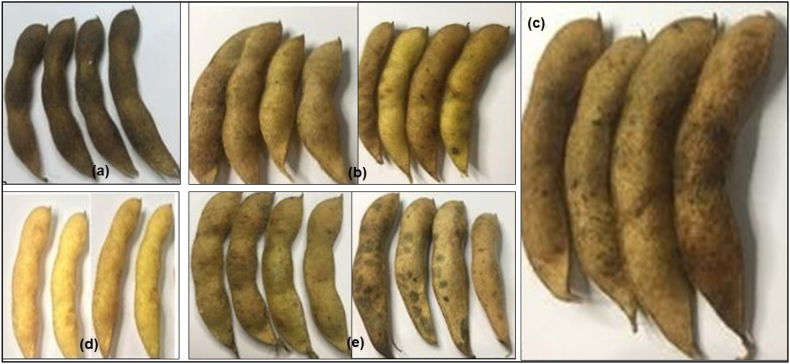
The color of the pods of several soybean varieties in South Sulawesi: (a) dark brown pods (Deja-1 variety); Brown pods (Dena-2 and Devon-2 varieties); (c) yellowish brown pods (Dena-1 variety); (d) yellow pods (Devon-1 variety); (e) light brown pods (Deja-2 and Dega-1 varieties).

The skin color of the soybean pods of the 13 varieties studied was found to be 5 pod colors namely dark brown, brown, yellowish brown, yellow, and light brown (Fig. 4). In addition to the color of the brown pods, several varieties were light yellow and cream in color. Varieties that have brown pods were Dena-2 and Devon-2, dark brown are Deja-1, Gepak Kuning, Argomulyo, Grobogan, yellowish brown pods was Dena-1. While the color of the yellow pods were Devon-1, Derap-1, Detap-1, the color of the pods was light brown were Demas-1, Deja-2, Anjasmoro, Dega-1 [11] Meanwhile, according to Ref. [17] the Grobogan variety has brown pods, Anjasmoro has light brown pods, and Gepak Kuning has brown pods.

### 3.6 Number of pods per node

Soybean plants have nodes starting from the first node, second node, third node, fourth node, and fifth node. In Table 4 it can be seen that the number of pods in the first node, of the 13 varieties tested, only 3 varieties had the same number of pods, namely Deja-1, Anjasmoro, and Dega-1 and the others had different numbers. In the second node, only one variety had the highest number of pods, Dena-2 and this was significantly different from 6 varieties, namely Detap-1, Deja-2, Dega-1, Grobogan, Dena-1, and Deja-1. The level of variation in the number of pods in the second node was lower than in the first node. In the first node the number of pods is more diverse, while in the second node it is more diverse. The number of pods in the third node of all the varieties tested varied greatly and only 2 varieties had a significantly different number of pods, the Demas-1 and Dega-1 varieties. Likewise, in the fourth node, the number of pods from the 13 varieties tested varied greatly and only 4 varieties had different numbers of pods, namely Demas-1 which was significantly different from Deja-2, Dega-1, and Grobogan. In the fifth node, the number of pods of all tested varieties was almost the same. Only 1 variety had the highest number of pods and was significantly different from the other 9 varieties. The number of pods formed is related to the presence of the number of fertile nodes which indicates that the variety can adapt to its environment. Differences in the number of fertile nodes are influenced by plant genetics which is determined by the variety of parents. Generally, taller soybean plants have more nodes so they will produce more pods [52]. The number of fertile nodes is related to the number of productive branches and affects production results [53]. Apart from plant genetic factors, environmental factors also play an important role in the formation of nodes and pods. High temperatures influence the formation of fertile nodes and the elongation of the nodes [54]. An increase in temperature results in fewer pods and seeds being formed and the yield index decreases [55]. The ability of plants to form pods and seeds is strongly influenced by the nutrient status of the soil. Nutrients absorbed from the roots will flow to all parts of the plant body, especially the leaves for the photosynthesis process so that the plants will utilize photosynthates in growth and the formation of pithy pods. The availability of element N is necessary for the formation of pods [56].

**Table 4:** Number of pods per node on several soybean varieties in South Sulawesi.

	Number of pods per node				
Type of variety	1^St^ Node	2nd Node	3rd Node	4th Node	5th Node
Derap-1	5.67bcd	5.33 ab	4.22abc	3.89 ab	3.55a
Devon-2	4.67abcd	5.56 ab	5.11abc	4.78abc	5.22 ab
Deja-1	2.67a	3.11a	3.89 ab	4.44abc	4.56a
Anjasmoro	3.22a	5.00 ab	4.89 abc	3.89 ab	6.78b
Dena-2	5.56bcd	7.22b	5.56BCE	5.22abc	4.22a
Dena-1	4.89abcd	3.89a	5.11abc	4.44abc	4.22a
GepakKuning	4.78abcd	5.33 ab	5.56BCE	4.00 ab	5.00 ab
Grobongan	3.44 ab	3.89a	3.56 ab	3.44a	3.67a
Devon-1	5.78cd	5.56 ab	5.22abc	6.11BCE	3.67a
Dega-1	3.00a	4.11a	2.78a	3.11a	3.44a
Deja-2	3.56 abc	4.00a	4.78abc	3.33a	3.33a
Demas-1	6.33d	5.11 ab	6.67c	6.55c	5.44 ab
Detap-1	4.44abcd	4.44a	4.22abc	4.22abc	4.44a

*The numbers in the same column followed by same letter are not significantly different according to Duncan's test at the 0.05 level.

### 3.7 The number of pest populations and the level of damage pests and diseases of soybean leaves and pods

The table shows lower locust population levels in Devon-2, Dena-2, Grobogan, Devon-1, and Dega-1 varieties, while green stink bug populations were also lower in Derap-1, Dena-1, Dena-2, Grobogan, and Devon-1 varieties, then leaf beetle population was lower in Devon-2, Dena-2 and Detap-1 variety. The *Valanga* sp damage was lower in Grobogan and Dega-1 varieties, while green stinkbug damage was lower in Devon-2, Dena-2, Derap-1, and Detap-1 varieties. Furthermore, the level of damage by leaf beetle pests was lower in Dena 2, Grobogan and Dega 1 (Table S1). Population levels and pest attacks on soybeans are very dependent on the availability of abundant food and the increase in soybean biomass [57].

The lowest population of *N. viridula* was found in the Devon-1 variety (2.33 individuals per 15 m^2^) and the highest in Anjasmoro (6.67 individuals per 15 m^2^). The results of [58] showed that the lowest population of *N.viridula* was found in the Devon-1 variety (3.87 individuals per 6 m^2^) and the highest in the Dega-1 variety (5.53 individuals per 6 m^2^). Table S1 shows that the lowest level of pod damage due to *N. viridula* attack was found in Grobogan (3.67 %), Detap-1 (3.45 %), Derap-1 (3.47 %), Devon-1 (3.15 %), and Dena-2 varieties (3.17 %), while the highest was Gepak kuning, Anjasmoro (12.78 %), and Deja-2 (12.16 %) varieties. The results of the study [59] revealed that the lowest attack rate of *N. viridula* on soybean pods was found in Grobogan (8.50 %), Argomulyo (9.40 %), and Detam-1 (9.0 %), while the highest was in Burangrang (13.20 %), Gema (12.50 %), and Dering-1 (10.50 %).

The level of damage by green stink bugs (*N. viridula*) and leaf beetles (*P. inclusa*) is strongly influenced by the morphology of soybean plants, especially the pods. The results of the study by Ref. [60] showed that the morphological characteristics of soybean plants in the form of pod shell thickness had a strong relationship with the intensity of the brown stink bug *(R. linearis*) attack. The soybean phenology influenced stink bug population dynamics, mainly in the inflorescence emergence, flowering, development of fruits and seeds, and ripening of fruits and seeds stages (71).Three varieties were resistant to stink bugs, namely Gepak Kuning, Seulawah, and Sinabung. Argomulyo was the only variety that had a category as highly susceptible. Other varieties were moderately resistant (Argopuro, Cikuray, Dering 1, Gumitir, Kaba, Merbabu, Sibayak, Tampomas, and Tanggamus) In Probolingo, East Java [61].

### 3.8 Seed hilum color

The color diversity of the hilum of soybean seeds observed in this study was soybean accessions with yellow-brown hilum (Fig. S1). Generally, soybean accessions that were characterized had seeds with brownish-yellow hilum. According to Ref. [62], the gene that controls hilum color is allelomorphic. The use of microsatellite markers shows that variations in the color or tone of the soybean hilum do not always correspond to genetic variations [63].

According to Ref. [64], the Grobogan variety has a brown helium color and Anjasmoro has a brownish-yellow hilum color. Furthermore, the Dega-1 variety has a brown hilum, Detap-1 was yellow, Demas-1 was dark brown, Dena-2 was brown, Devon-1 was light brown, Deja-1 was Light brown, Gepak was yellow-brown, Deja-2 was brown, Derap-1 Light brown, and Dena-1 brown [20].

### 3.9 Aspects of processing soybeans into products

Soybean is a type of edible legume with tremendous nutritional value in terms of protein content. There are many types of soybean utilization worldwide, especially in Asia and the United States. Soybeans are used in manufacturing, soy oil, soy milk, tofu, tempeh, fermented bean paste, soy sauce, soy flour, meat analogs and milk, soy butter, vodka, and many other products. Soybean oil is the most widely produced and consumed vegetable oil in the world. Soybean oil is cholesterol free like all vegetable oils and contains high amounts of unsaturated fats. Therefore soybean oil which is 20 % of soybean seeds is used to make salads, as cooking oil, and as margarine. The high content of linoleic acid makes soybean oil more important than other animal-based vegetable and vegetable oils [65].

Indonesia has an excellent opportunity to increase soybean production and meet domestic demand. This opportunity can be seen from market demand, land availability and better varieties, and the government's strong will. The demand for soybeans as food and feed is increasing continuously and is expected to increase in the coming years. The highest-demand portion comes from processed foods, especially tempeh and tofu. Another high demand comes from the animal feed industry which is expected to continue to increase as part of the increase in cattle production. Therefore, by increasing national soybean production, the government wants to meet this demand by using national production and reducing imports [66].

Based on the color of the seeds, yellow soybeans and black soybeans are known. Black soybeans are generally only used as raw material for soy sauce, while yellow soybeans are used as raw materials for tempeh, soy milk, tofu, and other foods (tauco and others) [67].To meet the needs of the soybean-based industry, several high-yielding soybean varieties released recently have diverse properties. Generally, these varieties have large and yellow seeds, such as Argomulyo, Bromo, Burangrang, Panderman, Anjasmoro, and Grobogan whose seed sizes are the same, even more, significant than imported soybeans, and have higher protein content than imported soybeans or the Wilis variety which has been had long been cultivated by farmers [68].

Soybean is the primary raw material for making soy milk. Soy milk can be obtained by soaking soybeans, milling soybeans using water, and filtering soybean pulp. Different types of soybeans can affect the chemical and sensory composition of the resulting soy milk, such as protein content, total solids, and beany flavor attributes. The best type of soybean used in the manufacture of soy milk is US soybean because it has sensory quality, especially the beany flavor which is lower than the other four types of soybeans, as well as high viscosity, although the yield of soy milk produced is lower than other types of soybeans [69].

### 3.10 Stove weight, the weight of 100 seeds, and seed yield

#### 3.10.1 Production of soybean stover

The results showed that the potential for soybean straw was very high, as seen from the average production of all varieties above 50 % of seed production (Table S2), even higher than that reported [*80*], where the ratio between seeds with soybean straw is 1.5 kg per 1 kg of seeds.

The table above shows that the height of soybean plants varies from each variety, where the Dena-2 variety was the lowest plant compared to other plants, namely 20.8 cm, and was very significantly different from other varieties and the highest was the Devon-2 variety reaching 33. 67 cm, although not significantly different from the Detap-1 variety. Plant growth can be influenced by a variety [ [70,71]]. In each plant variety, there are always differences in response to various environmental conditions in which it grows [72]. This situation makes the difference in the growth and production of each variety, so the plant height and number of branches may be different for each variety. The Gepak Kuning and Dena-1 varieties, and the one with the most stalks was the Gepak Kuning variety, significantly higher than the Deja-1, Anjasmoro, Grobogan and Devon-1 varieties, but not significantly different from the other varieties. Even though the height and the number of branches were many, they were not positively correlated with the branch weight of each soybean plant variety, whereas the Gepak kuning variety with the highest number of branches has the lowest stem weight.

#### 3.10.2 Weight 100 seeds

The weight of 100 seeds from the 13 varieties tested, there were 4 varieties that had seed sizes between 24.67 and 27.72 g per 100 seeds and there were 8 varieties that had seed sizes of 15.69–23.79 g per 100, but only 1 variety had a size of 10.96 g per 100. The seed size characteristics of each variety are greatly influenced by the genes of each variety. This can be seen from the large seed size of each variety in the Description of Varieties [11], the seed size of the Gepak Kuning variety is only 8.25 g, Grobogan 18 g, Anjasmoro 14.8–15.3 g, Argomulyo 16.0 g, Dema-1 14.99 g, Dena-2 13.0 g, Dena-1 14.3 g, Dega-1 22.98 g, Demas-1.13 g, Derap-1 17.62 g, Detap-1 15.37 g, Deja-1 12.39 g, and Deja-2 14.8 g per 100 seeds. The seed size of each variety is a reflection of the size of the seeds of each variety. The size of the seeds is one of the factors that influences the selling value of a variety. Large-sized soybeans have the opportunity to have a higher price per kilo compared to varieties that have small seed sizes.According to Ref. [73] that large-seeded soybeans will produce seeds per plant, pods per plant, and maximum seed yield per plant, because of the presence of reserves. food reserves stored in large seeds lead to high germination vigor index [70]. Seed size is important for optimizing yield and also plays an important role in the condition of a cultivar and influences seed vigor [71]. Furthermore [72], suggested that seed length, seed width, and seed height were characteristics of seed size and their comparison as characteristics of seed shape for 184 recombinant inbred lines and 219 successful cultivated accessions. They reported that the seed size trait, which is controlled by multiple genes in soybean, plays an important role in determining seed yield, quality, and appearance. Regarding soybean seed traits [74], reported that direct selection for a greater number of seeds per plant and higher seed yield per plant would result in greater genetic improvements in seed yield.

From the results of [52], it was found that the genotype G511H/Anjs-8-5 yielded 3.08 tha^-1^, early maturity (75 days), and large seed size (16. 92 g per 100 seeds) has the potential to be developed in Indonesia's tropical climate area and support the supply of raw materials for the tempe industry. In this research, varieties that have the potential to be raw materials for tempeh were varieties Dena 2 (27.72 g per 100 seeds), Dega 1 (25.93 g per 100 seeds), Detap 1 (25.25 g per 100 seeds), Grobogan (24. 67), Derap 1 (23.79 g per 100 seeds), Deja 1 (22.29 g per 100 seeds), Anjasmoro (20.33 g per 100 seeds), Dena 1 (19.83 g per 100 seeds), Devon 1 (18.46 g per 100 seeds), Demas 1 (16.61 g per 100 seeds).

#### 3.10.3 Seed yield

The highest seed yields produced from several soybean varieties were the Dena-2 variety (3.78 t ha^−1^) and the lowest were the Anjasmoro varieties (1.93 t ha^−1^) and Deja-2 (2.02 %), while the Grobogan seed yields achieved were around 2.96 t ha^−1^ (Table S2). The results of the study [*14*] showed that the Anjasmoro variety had higher seed yields (13.24 g plant^−1^) than the Grobogan variety (12.55 g plant^−1^). Furthermore, the results of research [13], the seed yield of the Anjasmoro variety was higher (18.06 g plant^−1^ or 2.71 t ha^−1^) than the Grobogan variety (17.03 g plant^−1^or 2.10 t ha^−1^). The results of research by Fattah et al. [73] in Pangkep Regency, the seed yields obtained at the Anjasmoro variety were higher (2.71 t ha^−1^) than Grobogan (2.10 t ha^−1^). While the Dena-2 variety has several advantages, including lodging resistance, pods not breaking easily, high seed yield potential, resistance to pod-sucking pests, and *S. litura* armyworm pests [11].

### 3.11 Market opportunities for several soybean varieties in South Sulawesi

Soybean commodity marketing activities are a bridge between producing farmers and soybean traders, tofu and tempeh craftsmen, and other processing industries such as the soy sauce industry [75]. Producing farmers have some alternatives in selling the soybeans they produce, although the traders who operate up to the farm level are only village collector traders, inter-village collectors, and sub-district traders they are numerous and provide a variety of prices received by farmers. According to Ref. [76] disclosed that soybeans and processed products such as tempe are 'low-cost nutritious food' which are consumed by all socio-economic groups in Indonesia and Malaysia. The results of [77] revealed that the soybean seeds that are in demand by tempeh producers are those that are yellow in color, have large seed sizes, and have thin seed skins because they can produce tempeh that is bright in color and has large volume (bloom). In line with [63] stated that seed size determines the quality of tempe because it has a positive correlation with the weight and volume of tempeh. Until now, tempe producers still use imported soybeans for several reasons including lower prices, abundant availability in the market, uniform color quality and bean size, the seeds are not mixed with feces, and when processed into tempeh, the yield of tempeh becomes larger [78].

In general, local soybeans are small to medium-sized seeds so the average yield of tempeh from local soybeans is 25 % lower than imported soybeans. In addition, the condition of local soybeans is usually less clean and must be sorted again so the consequences will be additional costs per labor for the sorting process and a reduction in the percentage of seeds per unit weight [79]. This is in line with the results of research that has been carried out in South Sulawesi that several soybean varieties have the performance that is favored by tempeh producers, including large and yellow seeds. These varieties include Devon −2, Anjasmoro, Grobogan, and Devon-1 and have a productivity of more than 2 tonnesha^−1^. So that the soybean superior varieties have the opportunity in marketing.

## 4 Conclusion

1.Based on the performance and characteristics of the 13 soybean varieties above, it can be concluded that: there are 4 varieties that have the highest plant height in the vegetative phase, namely Devon-2 and Detap-1 and in the vegetative vase Gepak Kuning and Detap-1. The shape of soybean leaves was found in 3 varieties with slightly round leaves, 2 varieties with round leaves, 6 varieties with oval leaves, 1 variety has triangular leaves, and 1 variety has sharp leaves. Nitrogen content, there are 3 varieties that have a nitrogen content between 11.0 and 12.64 and 10 varieties that have a nitrogen content between 9.05 and 10.65. Leaf greenness level, there is 1 variety that has the highest leaf greenness level (31.59), 6 varieties that have leaf greenness levels between 25.17 and 27.35, and 6 varieties that have leaf greenness levels between 19.69 and 23.67. Leaf area, there are 5 varieties that have a leaf area between 3.50 and 4.15 and 8 varieties that have a leaf area between 2.90 and 3.49. Pod skin color, there are 4 varieties yellow/yellowish brown and 9 varieties brown/light brown. Hilum color, there are 10 brown varieties and 3 yellow/brownish varieties. Seed size, there are 5 varieties that have the largest size (Dega-1, Grobogan, Derap-1, Dena-2, and Devon-2). These 5 varieties have the opportunity for the highest price per kilogram, because people like the largest seed size soybeans. Straw weight, there are 3 varieties that have the highest weight (Derap-1, Deja-1, and Dena-1). Of the three varieties, straw has a very big opportunity to be used as animal feed.

2. Response to pests and diseases of the 13 varieties tested are *Valanga* sp pest population levels, there are 3 varieties that respond with the highest populations (Anjasmoro, Demas-1, and Deja- 2). The population level of the *N.viridula* pest, there were 3 varieties that responded with the highest population (Anjasmoro, Demas-1, and Deja-2) and 3 varieties with the lowest (Devon-1, Dena-2, and Derap-1). Population level of *P. inclusa*, there was 1 variety that responded with the highest population (Anjasmoro) and there were 7 varieties with the lowest response. The level of leaf damage due to Valangasp attack, there were 2 varieties that responded with the highest leaf damage symptoms (Demas-1 and Anjasmoro). The level of pod damage due to attack by the pod-sucking pest *N.viridula*, there were 2 varieties that responded with the highest level of pod damage (Anjasmoro and Deja-2 and the 2 lowest varieties (Devon-2 and Dena-2). The level of leaf damage due to attack P. inclusa pest, there were 3 varieties that responded with the highest level of pod damage (Anjasmoro, Gepak Kuning, and Deja-2) and 3 varieties the lowest (Dena-2, Grobogan, and Dega-1). The level of damage to soybean seeds due to attack *Cercospora*sp disease, there was 1 variety that responded with the highest level of damage (Gepak Kuning) and there was 1 variety that had the lowest level of damage (Dena-2).

3.Seed yield achieved by each variety, there are 3 varieties that give the highest seed yield Dena-2 (3.78 t ha-1), Devon-2 (3.31 t ha-1), and Derap-1 (3.13 t ha-1) and there is 1 variety that gives the lowest seed yield Anjasmoro (1.93 t ha-1) and Deja-2 (2.02 t ha^−1^).

## Informed consent Statement

Informed consent letters were obtained from all subjects involved in learning.

## Data availability Statement

Not applicable.

## Conflect of interest

The authors declare no conflect of interest.

## CRediT authorship contribution statement

**Abdul Fattah**: Conceptualization, Methodology, Writing-original draft, Validation, Data Curation, Supervision, Resources. **Idaryani**: Conceptualization, Methodology, writing-original draft, validation, funding acquisition, resources, Formal analysis. **Herniwati**: Methodology, writing-original draft, Software, Funding acquisition, Resources, Data curation, Formal analysis. **M. Yasin**: Conceptualization, Methodology, Writing-original draft, Validation, Data Curation, Supervision, Resources, Formal analysis. **Suriani Suriani**: Conceptualization, Investigation, Methodology, Writing – original draft, Writing – review & editing, Visualization, Formal analysis. **Salim**: Investigation, Methodology, Writing – original draft, Writing – review & editing, Data curation, Visualization, Formal analysis. **M. Basir Nappu**: Data curation, Methodology, writing-original draft, Software, Funding acquisition, Resources, Data curation. **Sahardi Mulia**: Data curation, Software, Funding acquisition, methodology, writing-original draft, supervision, formal analysis. **Muh Fitrah Irawan Hannan**: writing-original draft. **Heppy Suci Wulanningtyas**: writing-original draft. **Sudjak Saenong**: Writing – review & editing. **Wanti Dewayani**: Copceptualization, investigation, methodology, software, funding acquisition, resources, formaly analysis. **Suriany**: Writing – review & editing. **Elisa Winanda**: Writing – review & editing. **Sri Wahyuni Manwan**: Investigation, Methodology, Writing – original draft, Writing – review & editing, Visualization, Data curation, Formal analysis. **Muh. Asaad**: Data curation, Software, Funding acquisition, methodology, writing-original draft, supervision, formal analysis. **Warda**: Conceptualization, Methodology, Writing-original draft, Validation, Data Curation, Supervision, Resources. **Nurjajani**: Data curation, Methodology, writing-original draft, Software, Funding acquisition, Resources, Data curation. **Nurhafsah**: Writing-original draft. **Abdul Gaffar**: Visualization. **Sunanto**: Investigation, Methodology, Writing– original draft, Writing – review & editing. Conceptualization, Visualization, Formal analysis. **Andi Yulyani Fadwiwati**: Writing – review & editing. **Maryam Nurdin**: Writing – review & editing. **Dahya**: Writing – review & editing. **Andi Ella**: Conceptualization, Methodology, Writing-original draft, Validation, Data Curation, Supervision, Resources.

## Declaration of competing interest

The authors declare that they have no known competing financial interests or personal relationships that could have appeared to influence the work reported in this paper.

## References

[1] Ministry of Trade (2022).

[2] Fattah A., Syam S., Daud I.D., Sartika Dewi V., Rahman A. (2018). The intensity of leaf damage caused by attack of Spodoptera litura F and seed yield on some soybean varieties in South Sulawesi Indonesia. Scientific Research Journal (SCIRJ).

[3] Krisdiana R. (2013). Distribution of superior varieties of soybeans and their impact on the rural economy. Journal of Agricultural Research Food Crops.

[4] Abdul Fattah, Sjam Sylvia, Itji Diana Daud, SartikaDewi Vien (2018). The relationship of the population density of Larvae Spodoptera litura with the leaf damage and decrease of seed yield for soybean, Indonesia. J. Agric. Sci. Technol..

[5] Fattah A., Djamaluddin I., Ilyas A., Muslimin, Nurhayu A., Yasin M. (2022).

[6] Hafid H., Syaiful S.A., Kaimuddin, Fattah A., Djufry F. (2021).

[7] Fattah A., Winanda E., Salim, Sri W., Idaryani M., Dewayani W. (2023).

[8] Marwoto, Sri Hardaningsih, Abdullah Taufiq (2013).

[9] Shilpashree N., Devi S.N., Manjunathagowda D.C., Muddappa A., Abdelmohsen S.A.M., Tamam N. (2021). Morphological characterization, variability and diversity among vegetable soybean (Glycine max L.) genotypes. Plants.

[10] Nawaz M.A., Lin X., Chan T.F., Ham J., Shin T.S., Ercisli S. (2020).

[11] Indonesian Legume, Tuber Crops Research Institute, I (2016).

[12] Hanafiyanto F., Wahono (2021). Comparison of the accuracy of measuring chlorophyll and nitrogen levels between SPAD and NDVI in corn plants (Zea mays). Indragiri Agro Journal.

[13] Apriani D., Supeno B., Haryanto H. (2020). Host preference test for Spodoptera frugiperda on several food crops. SAINTEK Proceedings.

[14] Ningsih F., Zubaidah S., Kuswantoro H. (2017). Proceedings of the National Seminar on UM Postgraduate Science Education.

[15] (2019). Syariani Br Tambunan; Afkar. Growth of Varieties of Soybean (Glycine Max L. Merrill) on Ultisol Soil of Southeast Aceh District.

[16] Zainuddin R., Yusuf N M., Usnawiyah U., Ismadi I., Nazaruddin M. (2022). Morpho-physiological adaptation test of several varieties of soybean (Glycine max.L) due to shade level treatment. Journal of Scientific Agroecotechnology Students.

[17] Suhartini, Purwantoro, Aufiq A., Nugrahaeni N. (2013).

[18] Fattah A. (2018).

[19] Gong W., Qi P., Du J., Sun X., Wu X., Song C. (2014). Transcriptome analysis of shade-induced inhibition on leaf size in relay intercropped soybean. PLoS One.

[20] Hasani R., Mehregan I., Larijani K., Nejadsattari T., Scalone R. (2017). Survey of the impacts of soil and climatic variations on the production of essential oils in Heracleum persicum. Biodiversitas.

[21] Poorter H., Niinemets Ü., Ntagkas N., Siebenkäs A., Mäenpää M., Matsubara S. (2019). A meta-analysis of plant responses to light intensity for 70 traits ranging from molecules to whole plant performance. New Phytologist. Blackwell Publishing Ltd August 1.

[22] Sadeghi S.M., Ali S., Niyaki N. (2013). Effects OF planting date and cultivar on the yield and yield components of soybean in north of Iran. ARPN Journal of Agricultural and Biological Science.

[23] Wu Y., Gong W., Wang Y., Yong T., Yang F., Liu W. (2018). Leaf area and photosynthesis of newly emerged trifoliolate leaves are regulated by mature leaves in soybean. J. Plant Res..

[24] Srihartanto E., Indradewa D. (2019). Effects of planting time and cultivar on leaf physiology and seed yield of soybean (Glycine max. (L.) merr). Caraka Tani: J. Sustain. Agric..

[25] Jin J., Li Y., Liu X., Wang G., Tang C., Yu Z. (2017). Elevated CO2 alters distribution of nodal leaf area and enhances nitrogen uptake contributing to yield increase of soybean cultivars grown in Mollisols. PLoS One.

[26] Zhong H., Liu S., Sun T., Kong W., Deng X., Peng Z. (2021). Multi-locus genome-wide association studies for five yield-related traits in rice. BMC Plant Biol..

[27] Farid M., Shakoor M.B., Ehsan S., Ali S., Zubair M., Hanif M.A. (2013). Morphological, Physiological and Biochemical Responses of Different Plant Species to Cd Stress.

[28] Shi H., Guo J., An J., Tang Z., Wang X., Li W. (2023). Estimation of chlorophyll content in soybean crop at different growth stages based on optimal spectral index. Agronomy.

[29] Liu Y., Li M., Xu J., Liu X., Wang S., Shi L. (2019). Physiological and metabolomics analyses of young and old leaves from wild and cultivated soybean seedlings under low-nitrogen conditions. BMC Plant Biol..

[30] Su B.Y., Song Y.X., Song C., Cui L., Yong T.W., Yang W.Y. (2014). Growth and photosynthetic responses of soybean seedlings to maize shading in relay intercropping system in Southwest China. Photosynthetica.

[31] Li Y., He N., Hou J., Xu L., Liu C., Zhang J. (2018). Factors influencing leaf chlorophyll content in natural forests at the biome scale. Frontiers in Ecology and Evolution.

[32] Houborg R., McCabe M., Cescatti A., Gao F., Schull M., Gitelson A. (2015). Joint leaf chlorophyll content and leaf area index retrieval from Landsat data using a regularized model inversion system (REGFLEC). Remote Sensing of Environment.

[33] Wu Y. wei, Li Q., Jin R., Chen W., Liu X. lin, Kong F. lei (2019). Effect of low-nitrogen stress on photosynthesis and chlorophyll fluorescence characteristics of maize cultivars with different low-nitrogen tolerances. J. Integr. Agric..

[34] Puja Santana F., Ghulamahdi M., Lubis I. (2020). ResponsPertumbuhan, fisiologi, dan ProduksiKedelaiterhadapPemberianPupuk nitrogen denganDosis dan waktu yang berbeda. JurnalIlmuPertanian Indonesia.

[35] Onoda Y., Wright I.J., Evans J.R., Hikosaka K., Kitajima K., Niinemets Ü. (2017). Physiological and structural tradeoffs underlying the leaf economics spectrum. New Phytol..

[36] Sodiq M. (2009).

[37] Xue F., Liu W., Cao H., Song L., Ji S., Tong L. (2021). Stomatal conductance of tomato leaves is regulated by both abscisic acid and leaf water potential under combined water and salt stress. Physiol. Plantarum.

[38] Patel P., Jatav P.K., Jain R., Gupta S., Kothari S.L., Kachhwaha S. (2021). Humidity induced opening of stomata leads to enhanced uptake of copper nanoparticles in triticumaestivum l. Mater. Today: Proceedings; Elsevier Ltd.

[39] Lysenko E.A., Kozuleva M.A., Klaus A.A., Pshybytko N.L., Kusnetsov V.V. (2023). Lower air humidity reduced both the plant growth and activities of photosystems I and II under prolonged heat stress. Plant Physiol. Biochem..

[40] Zahra N., Hafeez M.B., Ghaffar A., Kausar A., Zeidi M. Al, Siddique K.H.M. (2023). Plant photosynthesis under heat stress: effects and management. Environmental and Experimental Botany. Elsevier B.V. February.

[41] Feng X., Liu R., Li C., Zhang H., Slot M. (2023). Contrasting responses of two C4 desert shrubs to drought but consistent decoupling of photosynthesis and stomatal conductance at high temperature. Environ. Exp. Bot..

[42] Sakoda K., Watanabe T., Sukemura S., Kobayashi S., Nagasaki Y., Tanaka Y. (2019). Genetic diversity in stomatal density among soybeans elucidated using high-throughput technique based on an algorithm for object detection. Sci. Rep..

[43] Lundgren M.R., Mathers A., Baillie A.L., Dunn J., Wilson M.J., Hunt L. (2019). Mesophyll porosity is modulated by the presence of functional stomata. Nat. Commun..

[44] Wu Z., Chen L., Yu Q., Zhou W., Gou X., Li J. (2019). Multiple transcriptional factors control stomata development in rice. New Phytol..

[45] Zhang F., Ren F., Li J., Zhang X. (2022). Automatic stomata recognition and measurement based on improved YOLO deep learning model and entropy rate superpixel algorithm. Ecol. Inf..

[46] Siqueira J.A., Oliveira de Oliveira H., Nunes-Nesi A., Araújo W.L. (2021). Guard cell regulation: pulling the strings behind the scenes. Trends Plant Sci..

[47] Zhang Y., Wang P., Shao W., Zhu J.K., Dong J. (2015). The BASL polarity protein controls a MAPK signaling feedback loop in asymmetric cell division. Dev. Cell.

[48] Zhang Q., Peng S., Li Y. (2019). Increase rate of light-induced stomatal conductance is related to stomatal size in the genus Oryza. J. Exp. Bot..

[49] Caldera H.I.U., De Costa W.A.J.M., Woodward F.I., Lake J.A., Ranwala S.M.W. (2017). Effects of elevated carbon dioxide on stomatal characteristics and carbon isotope ratio of Arabidopsis thaliana ecotypes originating from an altitudinal gradient. Physiol. Plantarum.

[50] Torii K.U. (2021). Stomatal development in the context of epidermal tissues. Ann. Bot..

[51] Krisnawati A., Adie M.M. (2017). Variability on morphological characters associated with pod shattering resistance in soybean. Biodiversitas.

[52] Badiaraja P.H., Zubaidah S., Kuswantoro H. (2021). Maternal effect of agronomic and morphological characters on cluster structure of F3 soybean lines. Biodiversitas.

[53] Kuswantoro H. (2017). Genetic variability and heritability of acid-adaptive soybean promising lines. Biodiversitas.

[54] Allen L.H., Zhang L., Boote K.J., Hauser B.A. (2018). Elevated temperature intensity, timing, and duration of exposure affect soybean internode elongation, mainstem node number, and pod number per plant. Crop Journal.

[55] Burroughs C.H., Montes C.M., Moller C.A., Mitchell N.G., Michael A.M., Peng B. (2022). Reductions in leaf area index, pod production, seed size and harvest index drive yield loss to high temperatures in soybean. J. Exp. Bot..

[56] Puspasari R., Karyawati A.S., Sitompul s.M. (2018). Poll formation and yield of soybean (Glycine max (l.) Merrill) with provision of nitrogen in the generative phase establishment of pods and yield of soybean (glycine max (l.) Merrill) with the provisions of nitrogen on generative phase. Journal of plant production.

[57] Defensor M.O., Gonring A.H.R., Borges L.F., Plata-Rueda A., Martínez L.C., Fernandes F.L. (2020). Population dynamics of stink bugs (Heteroptera: pentatomidae) associated at various soybean phenological stages. J. Plant Dis. Prot..

[58] Kuswantoro H., Mejaya I.M.J., Baliadi Y. (2020). Determination of agronomical characters as the resistance attributes of twenty soybean varieties to stink bug (Nezaraviridula L.). Agrivita.

[59] Owen Maine F.V. (1928).

[60] Rabel M., Vieira E.S.N., Lana U.G. de P., Paiva E., Sehnem M.A.S., Schuster I. (2010). Marcadoresmolecularesmicrossatélitesnaavaliação de sementes de soja com variaçãonacoloração do hilo. Rev. Bras. Sementes.

[61] Hasbianto A., Hartati S., Weebadde C.K. (2020). Opportunities, challenges, and strategies to increase soybean production in Indonesia. JurnalInformasiTeknologiPertanian (JITP).

[62] Balanay R., Laureta R. (2021). Towards boosting the supply chain of soybeans for food security and import substitution in caraga region, Philippines. Journal of Ecosystem Science and Eco-Governance.

[63] Ginting E., Antarline S.S., Widowati S. (2009). Superior varieties of soybeans for raw materials for the food industry. Journal of Agricultural Research and Development.

[64] Marwoto A., Kutu Inayati (2011).

[65] Adawiyah D.R., Andarwulan N., Triana R.N., Agustin D., Gitapratiwi D. (2018). Evaluation of differences in soy bean varieties on the quality of soy milk products. Food Quality Journal.

[66] Gonzalez P.G.A., de Jesus Gariboti J.C., Leal Silva J.F., Lopes E.S., Abaide E.R., Lopes M.S. (2022). Soybean straw as a feedstock for value-added chemicals and materials: recent trends and emerging prospects. Bioenergy Research. Springer.

[67] Marliah A., Hidayat T., Husna N. (2012). The effect of variety and planting distance on the growth of soybean [Glycine Max (L.) Merrill]. J. Agrista.

[68] Chavan N.G., Bhujbal G.B., Manjare M.R. (2014). Effect of seed priming on field performance and seed yield of soybean [Glycine max (L.) Merill] varieties. Bioscan.

[69] Bakal H., Gulluoglu L., Onat B., Arioglu H. (2017). The effect of growing seasons on some agronomic and quality characteristics of Soybean varieties in mediterranean region in Turkey. Turkish Journal of Field Crops.

[70] Vidak M., Lazarević B., Javornik T., Šatović Z., Carović-Stanko K. (2022). Seed water absorption, germination, emergence and seedling phenotypic characterization of the common bean landraces differing in seed size and color. Seeds.

[71] Morrison M.J., Xue A.G. (2007). The influence of seed size on soybean yield in short-season regions. Can. J. Plant Sci..

[72] Hu Z., Zhang H., Kan G., Ma D., Zhang D., Shi G. (2013). Determination of the genetic architecture of seed size and shape via linkage and association analysis in soybean (Glycine max L. Merr.). Genetica.

[73] Adebisi M.A., Kehinde T.O., Salau A.W., Okesola L.A., Porbeni J.B.O., Esuruoso A.O. (2013). Influence of different seed size fractions on seed germination, seedling emergence and seed yield characters in tropical soybean {Glycine max L. merrill). Int. J. Agric. Res..

[74] Sudarić A., Vratarić M. (2002). Variability and interrelationships of grain quantity and quality characteristics in soybean. Bodenkultur.

[75] Saptana S. (1993). Agro Economic Research Forum.

[76] Dinesh Babu P., Bhakyaraj R., Vidhyalakshmi R. (2009). A Low Cost Nutritious Food “Tempeh”.

[77] Krisdiana R. (2018).

[78] Hidayah N., Setia Adiandri R., Astuti Mary, Bogor A., Agricultural Technology F., Major Postharvest Research and DevelopmentAgricultureJlnTentaraPelajar No, B (2012).

[79] Ginting E., Yulifianti R., Nutritional value and suitability of soybean varieties for tempeh. (*unpublished*). Teaching material of the Developing community work abilities and skills in the IHT environment through training on making tempe from various soybean varieties locally for tempe SMEs, Malang (September 29, 2015): 10 pp.

