# Supplementary material for: Performance and morphology of several soybean varieties and responses to pests and diseases in South Sulawesi
Source: Heliyon. 2024 Feb 2;10(5):e25507. doi: 10.1016/j.heliyon.2024.e25507 (PMC10907540; doi:10.1016/j.heliyon.2024.e25507)
Supplement: Multimedia component 1 [file mmc1.docx]

Supplementary file

**Performance and morphology of several soybean varieties and responses to pests and diseases in rainfed lowland rice fields**

**Table S1**. Number of pest populations and levels of pest and disease damage to soybeans

| Type of variety | Pest population  (individuals per 15 m^2^) | | | The intensity of damage to soybeans (%) | | | |
| --- | --- | --- | --- | --- | --- | --- | --- |
|  | *Valanga* sp (Acrididae) | *N. viridula* | *P. inclusa* | *Valanga* sp(leaf) | *N.viridula* (pod) | *P.inclusa* (leaf) | *Cercospora* sp (seed) |
| Derap-1 | 3.67a | 2.66a | 3.31ab | 8.63cde | 3.87ab | 5.63b | 8.01 d |
| Devon-2 | 2.66a | 3.33abc | 2.33a | 8.05bcd | 3.56a | 6.00b | 1.73ab |
| Deja-1 | 5.33b | 5.33de | 6.31cd | 11.97f | 6.24d | 6.99c | 2.16ab |
| Anjasmoro | 6.00bc | 6.67f | 7.33d | 21.03i | 12.18g | 12.84e | 2.31ab |
| Dena-1 | 3.00a | 2.60ab | 3.31ab | 8.57cde | 5.70cd | 5.69b | 3.14b |
| Dena-2 | 2.67a | 2.30a | 2.67ab | 7.78abc | 3.64a | 4.12a | 0.81a |
| Gepak Kuning | 5.33b | 6.30ef | 5.60c | 13.51g | 11.13f | 12.07de | 10.30e |
| Grobongan | 2.67a | 2.66ab | 3.31ab | 6.89a | 3.91ab | 4.69a | 5.91c |
| Devon-1 | 2.33a | 2.33a | 3.67b | 9.51e | 4.19ab | 6.00b | 3.00b |
| Dega-1 | 2.66a | 3.31abc | 3.32ab | 7.35ab | 4.82bc | 4.37a | 8.08d |
| Deja-2 | 6.33bc | 5.61ef | 6.61cd | 16.08h | 12.34g | 12.55de | 7.92d |
| Demas-1 | 7.00c | 4.30cd | 5.61c | 22.42i | 10.07e | 11.78d | 6.19cd |
| Detap-1 | 3.33a | 3.61bc | 2.67ab | 8.95de | 3.88ab | 5.95b | 5.86c |

*The numbers in the same column followed by same letter are not significantly different according to Duncan's test at the 0.05 level

**Table S2**. Seed and straw production of several soybean varieties in South Sulawesi

| Type of variety | Plant height (cm) | Number of Branches | Weight of straw (Kg ha^-1^) | Percentage of straw (%) | Weight of 100 seeds (g) | Seed Yield  (t ha^-1^) |
| --- | --- | --- | --- | --- | --- | --- |
| Derap-1 | 25,06bc | 3,67abc | 9681de | 67,63 | 23.79i | 3.13cd |
| Devon-2 | 33,67f | 3,47abc | 7813ab | 69,28 | 22.29h | 3.311d |
| Deja-1 | 28,40cde | 3,13ab | 10190e | 67,51 | 15.87c | 2.40b |
| Anjasmoro | 29,57de | 3,13ab | 8963cd | 78,43 | 20.33g | 1.93a |
| Dena-2 | 20,80a | 3,47abc | 8838cd | 73,35 | 19.83f | 3.78e |
| Dena-1 | 26,67bcd | 3,80bc | 10058e | 62,44 | 27.72m | 2.36b |
| GepakKuning | 26,60bcd | 4,00c | 6880a | 66,08 | 10.96a | 2.33b |
| Grobongan | 29,93de | 3,00a | 7260a | 59,29 | 24.67j | 2.96b |
| Devon-1 | 28,13bcd | 3,20ab | 7327a | 64,21 | 18.46e | 2.62b |
| Dega-1 | 27,93bcd | 3,33abc | 8513bc | 72,07 | 25.93l | 2.38b |
| Deja-2 | 24,80b | 3,40abc | 8717bcd | 76,80 | 15.69b | 2.02a |
| Demas-1 | 25,00bc | 3,53abc | 9269cde | 66,67 | 16.61d | 3.09cd |
| Detap-1 | 31,67ef | 3,27abc | 9497de | 72,62 | 25.25k | 2.60b |

Means followed by the same letter and same colom were not significantly different (P < 0.05).
