# Supplementary material for: Performance and morphology of several soybean varieties and responses to pests and diseases in South Sulawesi
Source: Heliyon. 2024 Feb 2;10(5):e25507. doi: 10.1016/j.heliyon.2024.e25507 (PMC10907540; doi:10.1016/j.heliyon.2024.e25507)
Supplement: Multimedia component 2 [file mmc2.docx]

**Supplementary file**

**Performance and morphology of several soybean varieties and responses to pests and diseases in rainfed lowland rice fields**


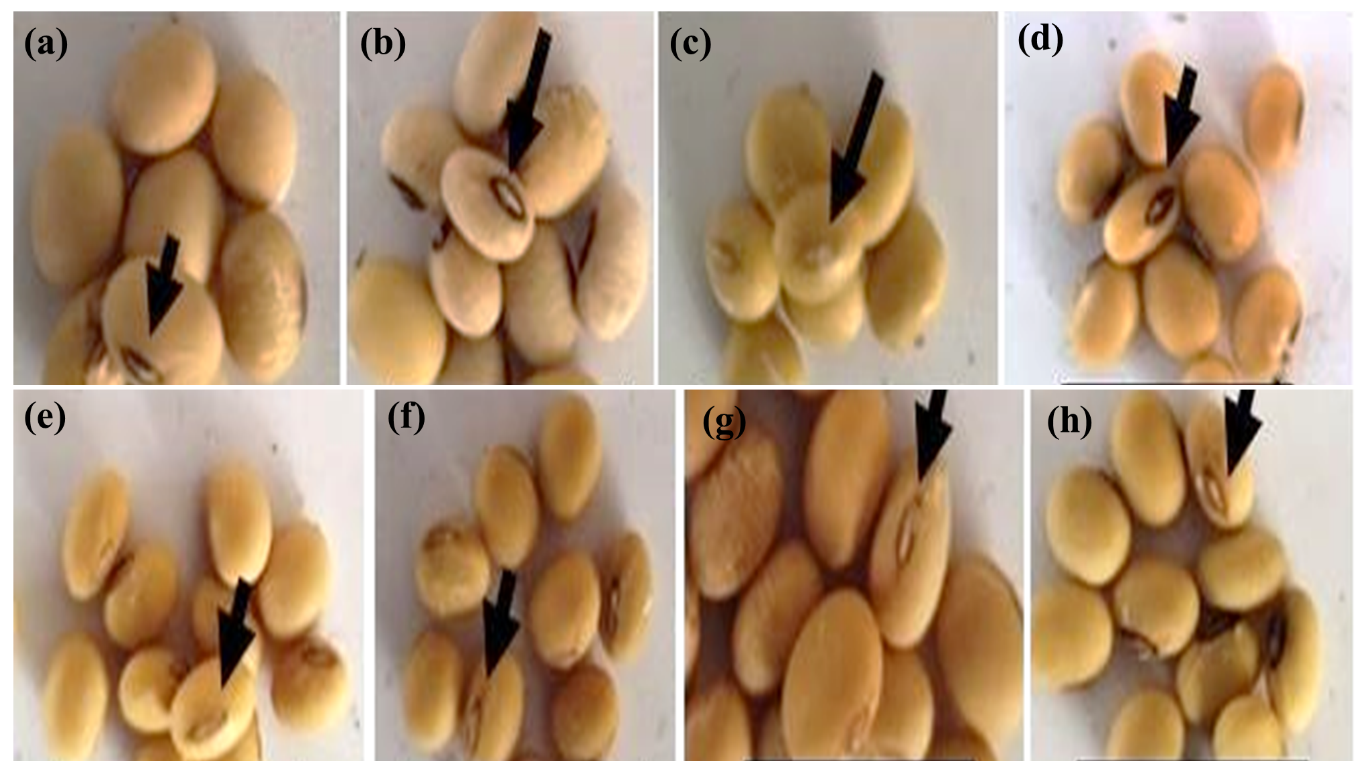


Figure S1. The color of the seeds hilum of several soybean varieties in South Sulawesi is generally brownish yellow. (a) Dega-1 variety; (b) Detap-1 variety; (c) Anjosmoro variety; (d) Demas Variety; (e) Dena-2 variety; (f) Devon-1 variety; (g) Grobogan Variety, and (h) Deja-1 variety.
